# The Role of Endoplasmic Reticulum Stress in Autoimmune-Mediated Beta-Cell Destruction in Type 1 Diabetes

**DOI:** 10.1155/2012/238980

**Published:** 2012-02-14

**Authors:** Jixin Zhong, Xiaoquan Rao, Jun-Fa Xu, Ping Yang, Cong-Yi Wang

**Affiliations:** ^1^The Center for Biomedical Research, Tongji Hospital, Tongji Medical College, Huazhong University of Science and Technology, 1095 Jiefang Avenue, Wuhan 430030, China; ^2^The Center for Biotechnology and Genomic Medicine, Medical College of Georgia, 1120 15th Street, CA4098, Augusta, GA 30912, USA; ^3^Affiliated Hospital of Guangdong Medical College, 57 Ren-Ming Road, Zhanjiang 524001, China; ^4^The Department of Clinical Immunology, Guangdong Medical College, 1 Xincheng Avenue, Dongguan 523808, China

## Abstract

Unlike type 2 diabetes which is caused by the loss of insulin sensitivity, type 1 diabetes (T1D) is manifested by the absolute deficiency of insulin secretion due to the loss of **β** mass by autoimmune response against **β**-cell self-antigens. Although significant advancement has been made in understanding the pathoetiology for type 1 diabetes, the exact mechanisms underlying autoimmune-mediated **β**-cell destruction, however, are yet to be fully addressed. Accumulated evidence demonstrates that endoplasmic reticulum (ER) stress plays an essential role in autoimmune-mediated **β**-cell destruction. There is also evidence supporting that ER stress regulates the functionality of immune cells relevant to autoimmune progression during T1D development. In this paper, we intend to address the role of ER stress in autoimmune-mediated **β**-cell destruction during the course of type 1 diabetes. The potential implication of ER stress in modulating autoimmune response will be also discussed. We will further dissect the possible pathways implicated in the induction of ER stress and summarize the potential mechanisms underlying ER stress for mediation of **β**-cell destruction. A better understanding of the role for ER stress in T1D pathoetiology would have great potential aimed at developing effective therapeutic approaches for the prevention/intervention of this devastating disorder.

## 1. Introduction

Recent epidemiologic studies revealed that the incidence of type 1 diabetes (T1D) in most regions worldwide has been increasing by 2% to 5% [1]. Particularly, in some developing countries such as China, the rapid economic development along with changes in lifestyle and presumably the living environment has rendered this country with an annual increase of 7.4% for T1D prevalence [2]. Given that T1D is typically developed in children and juveniles, its impact on the quality of life is far more significant than that of type 2 diabetes, in which it usually occurs in adults. Although exogenous insulin therapy partly compensates the function of *β* cells, it cannot regulate blood glucose as accurately as the action of endogenous insulin. As a result, long-term improperly control of blood glucose homeostasis predisposes T1D patients to the development of diverse complications such as diabetic retinopathy [3–5], nephropathy [6, 7], neuropathy [8–10], foot ulcers [11–13], and cardiovascular diseases [14–16]. Although the underlying mechanisms leading to T1D have yet to be fully addressed, extensive studies have consistently demonstrated that endoplasmic reticulum (ER) stress plays a critical role in autoimmune-mediated *β*-cell destruction during the course of T1D development.

The pancreatic *β*-cells are equipped with highly developed endoplasmic reticulum (ER) to fulfill the requirement of secreting a large amount of insulin. This physiological feature renders *β* cells particularly vulnerable to ER stress [17]. Exhaustion of *β* cells is essential for the onset of T1D, which requires the residual *β* cells for compensated insulin secretion. While this compensated action is beneficial for control of blood glucose homeostasis, it also increases ER burden associated with the induction of unfolded protein response (UPR) and ER stress, which further exacerbates *β*-cell death. Although the implication of ER stress in *β*-cell death has been extensively emphasized, the underlying mechanisms, however, are yet to be fully elucidated. As such, understanding the role of ER stress in the loss of *β* mass and dissecting the mechanisms underlying ER stress would be important for developing therapeutic approaches aimed at prevention and intervention of type 1 diabetes. In the present paper, we will first intend to address the overall role of ER stress in autoimmune-mediated *β*-cell destruction based on published genetic and experimental data. The impact of ER stress on modulation of autoimmune response during the course of T1D development will be next discussed. We will finally focus on the possible pathways implicated in the induction of ER stress and summarize the potential mechanisms underlying ER stress for mediation of *β*-cell destruction.

## 2. The Endoplasmic Reticulum (ER)

ER is a membranous network of tubules, vesicles, and cisternae that are interconnected by the cytoskeleton in the cytoplasm of eukaryotic cells. ER is responsible for many general cellular functions, including the facilitation of protein folding and assembly [18–20], manufacture of the membranes [21], biosynthesis of lipid and sterol, storage of intracellular Ca^2+^, and transport of synthesized proteins in cisternae.

ER can be categorized into rough endoplasmic reticulum (RER) and smooth endoplasmic reticulum (SER). RER is responsible for protein synthesis, while SER is in charge of the synthesis of lipids and steroids, regulation of calcium concentration, attachment of receptors on cell membrane proteins, and detoxification of drugs. As featured by its name, RER bears ribosomes on the outer surfaces of the cisternae and looks bumpy and rough under a microscope. The newly synthesized proteins by RER are sequestered in cisternae and sent to Golgi complex or membrane via small vesicles. In contrast, SER does not have ribosomes on its cisternae and appears to have a smooth surface under the microscope. SER is found commonly in places such as in the liver and muscle. It is important for the liver to detoxify poisonous substances. Sarcoplasmic reticulum (SR) is a special type of SER, which is found in smooth and striated muscle. SR is responsible for the regulation of calcium levels. It sequesters a large store of calcium and releases them when the muscle cell is stimulated.

## 3. ER Stress

**Figure 1:**
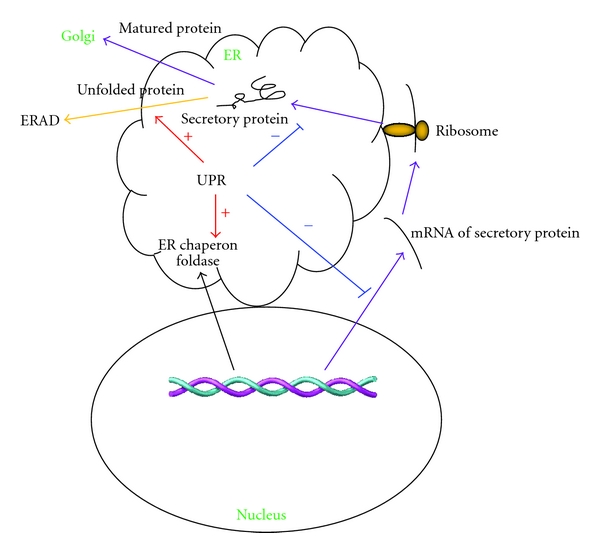
The regulatory role of unfolded protein response (UPR). Various physiological and pathological insults can result in the accumulation of unfolded proteins which then induces UPR and ER stress. In response to stressful insults, UPR regulates secretory pathway via following mechanisms: (1) enhancing (red arrow) the expression of ER chaperones and foldases to increase the folding capacity of ER; (2) attenuating (blue) the biosynthetic burden of secretory pathway through downregulating the expression of secreted proteins (purple arrow); (3) promoting the clearance of unfolded proteins by activating ERAD (orange arrow).

ER stress is the cellular responses to the disturbances of normal function of ER. The most focused and well-studied ER stress is that caused by protein misfolding. The accumulation of unfolded proteins leads to a protective pathway to restore ER function, termed as unfolded protein response (UPR). ER employs a type of special proteins called chaperones as a quality control mechanism. Chaperones attach to the newly synthesized proteins and assist them to fold into their native conformations. In addition, chaperones also help to break down unfolded or incorrectly folded proteins in the ER via a process called ER-associated degradation (ERAD). Protein folding requires a serial of ER-resident protein folding machinery. Exhaustion of those protein folding machineries or insufficient energy supply increases the accumulation of unfolded or misfolded proteins in ER, leading to the activation of UPR. Various physiological and pathological insults such as increased general protein synthesis, failure of posttranslational modifications, hypoxia, nutrient/glucose starvation, and alterations in calcium homeostasis can result in the accumulation of unfolded or misfolded proteins in ER which then causes ER stress [22]. For example, altered expression of antithrombin III [23, 24] or blood coagulation factor VIII [25, 26] results in the exhaustion of protein-folding machinery and thus induces UPR. Some physiological processes such as the differentiation of B lymphocytes into plasma cells along with the development of highly specialized secretory capacity can also cause accumulation of unfolded proteins and induce UPR [27–29]. In response to certain physiological and pathological insults, cells undergo UPR to get rid of the unfolded or misfolded proteins. Therefore, UPR is a protective mechanism by which it monitors and maintains the homeostasis of ER. For instance, UPR increases the folding capacity by upregulating ER chaperones and foldases, and attenuates the biosynthetic burden of secretory pathway through downregulating the expression of secreted proteins [30–32]. In addition, UPR also activates ERAD to eliminate unfolded proteins [33–35] (Figure 1). However, once the stress is beyond the compensatory capacity of UPR, the cells would undergo apoptosis. As such, UPR and ER stress are reported to be implicated in a variety of pathological processes, including diabetes, neurodegenerative diseases, pathogenic infections, atherosclerosis, and ischemia [22, 36].

As aforementioned, there is a monitoring mechanism to ensure the correct protein folding in ER. The unfolded proteins usually have a higher number of hydrophobic surface patches than that of proteins with native conformation [37]. Thus, unfolded proteins are prone to aggregate with each other in a crowed environment and directed to degradative pathway [38]. Molecular chaperones in ER are the major mechanisms to promote protein folding. They preferentially interact with hydrophobic surface patches on unfolded proteins and create a private folding environment by preventing unfolded proteins from interaction and aggregation with other unfolded proteins. In addition, the concentration of Ca^2+^ in ER also impairs protein folding by inhibiting the activity of ER-resident chaperones and foldases [39–42]. ER is the major site for Ca^2+^ storage in mammalian cells. The concentration of Ca^2+^ in ER is thousands times higher than that in the cytosol [43]. Most chaperones and foldases in ER are vigorous Ca^2+^ binding proteins. Their activity, therefore, is affected by the concentration of Ca^2+^ in ER. A variety of posttranslational modifications including N-linked glycosylation, disulfide bond formation, lipidation, hydroxylation, and oligomerization occur in ER. Disruption of those posttranslational modifications can also result in the accumulation of incorrectly folded proteins and thereby induce UPR or ER stress. For example, glucose deprivation impairs the process for N-linked protein glycosylation and thus leads to ER stress [44].

**Figure 2:**
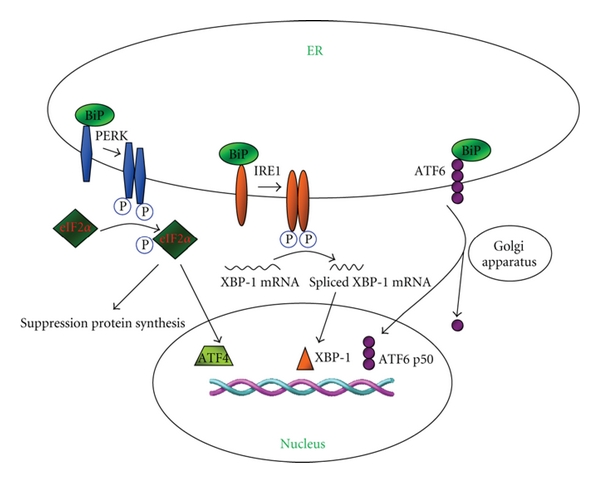
Signaling pathways relevant to UPR. PERK, IRE1, and ATF6 act as ER stress sensors by binding to the ER chaperone BiP, and by which they remain inactive under normal condition. Upon the accumulation of unfolded proteins, BiP preferentially binds to the unfolded proteins, which results in the release of PERK, IRE1, and ATF6. Once released from BiP, PERK becomes activated and dimerized. Activated PERK phosphorylates eIF2*α* to suppress the overall transcription of mRNAs while selectively enhance the transcription of genes implicated in UPR such as the ATF4 mRNA, and through which ATF4 initiates the transcription of UPR target genes. Similar to PERK, IRE1 is dimerized and activated after detached from BiP. IRE1 induces XBP-1 by promoting the splicing of its mRNA. XBP-1 activates the transcription of its target genes to enhance UPR. The release of ATF6 from BiP results in the translocation of ATF6 to the Golgi apparatus, where ATF6 is cleaved and then translocates into the nucleus, and by which ATF6 initiates the transcription of target genes.

UPR is mediated by three major pathways, which are initiated by the three transmembrane signaling proteins located on the ER membrane. Those transmembrane proteins function as a bridge to link cytosol and ER with their C-terminal in the cytosol and N-terminal in the ER lumen. The N-terminal is usually engaged by an ER-resident chaperone BiP (Grp78) to avoid aggregation. When unfolded proteins accumulate in ER, chaperons are occupied by unfolded proteins and release the transmembrane signaling proteins: which include the following three axes of signals: the pancreatic endoplasmic reticulum kinase (PERK), the inositol-requiring enzyme 1 (IRE1), and the activating transcription factor 6 (ATF6). The release of these proteins triggers UPR and ER stress (Figure 2). PERK is a Ser/Thr protein kinase uniquely present in ER. Once released from BiP, PERK becomes oligomerized and autophosphorylated. PERK inactivates eukaryotic initiation factor 2*α* (eIF2*α*) by phosphorylation of Ser51 to reduce mRNA translation and protein load on ER. Deficiency of PERK results in an abnormally elevated protein synthesis in response to the accumulation of unfolded proteins in ER. IRE1 is another axis of signal involved in UPR. IRE1 increases the production of X box protein-1 (XBP-1), a bZIP-family transcriptional factor, by promoting its mRNA splicing [45]. XBP-1 heterodimerizes with NF-Y and enhances gene transcription by binding to the ER stress enhancer (ERSE) and unfolded protein response element (UPRE) in the promoters of targeted genes. Unlike PERK and IRE1 which oligomerize upon UPR, when released from BiP, ATF6, the third axis of signal, translocates into the Golgi apparatus where its transmembrane domain is cleaved [46]. The cleaved ATF6 is then relocated into the nucleus to regulate the expression of targeted genes. For example, once released from the ER membrane, ATF6 enhances the transcription of XBP-1 mRNA which is further regulated by IRE1 [45].

## 4. ER Stress in Autoimmune-Mediated ***β***-Cell Destruction

Accumulative evidence supports that ER stress is implicated in autoimmune-mediated *β*-cell destruction in type 1 diabetes [47, 48]. It was noted that loss of *β* cells is the direct causing factor for insufficient insulin secretion in T1D patients. As described earlier, pancreatic *β* cells have a very well-developed ER to fulfill their biological function for secreting insulin and other glycoproteins, and therefore, *β* cells are highly sensitive to ER stress and the subsequent unfolded protein response (UPR). Severe or long-term ER stress would direct *β* cells undergoing apoptosis [47]. For example, mice deficient in PERK, a molecule responsible for regulating UPR, are extremely susceptible to diabetes. The *null* mice display a progressive loss of *β* mass and hyperglycemia with aging [49]. Consistent with the observations in these mice, some infant-onset diabetes in humans have also been confirmed to be associated with the mutations in PERK. For example, loss of *EIF2AK3* (the gene encodes PERK) develops Wolcott-Rallison syndrome, an autosomal recessive disorder characterized by early infancy insulin-dependent diabetes and multisystemic manifestations including growth retardation, hepatic/renal dysfunction, mental retardation, and cardiovascular abnormalities [50, 51]. Similarly, disruption of UPR by mutating eIF2*α*, a protein that controls mRNA translation upon ER stress, enhances the sensitivity to ER stress-induced apoptosis and results in defective gluconeogenesis. Mice carrying a homozygous Ser51Ala mutation for eIF2*α* show defective in pancreatic *β* cells manifested by the smaller core of insulin-secreting *β* cells and attenuated insulin secretion [52]. Altogether, defects in PERK/eIF2*α* signaling render *β* cells highly vulnerable to ER stress in both humans and mice [53, 54].

In type 1 diabetes, ER stress in the pancreatic *β* cells is primarily induced by proinflammatory cytokines produced by infiltrated immune cells, which then contributes to *β*-cell destruction. During the course of autoimmunity, pro-inflammatory cytokines are secreted by the infiltrated autoreactive immune cells in the milieu of pancreatic islets. For example, nitric oxygen (NO) is an inflammatory mediator resulted from autoimmune response during the course of type 1 diabetes. Studies have shown that excessive NO production induces *β*-cell apoptosis in a CHOP-dependent manner [55]. Other than ER stress caused by autoimmunity, misfolding of insulin in *β* cells can also directly induce chronic ER stress as evidenced by the observations in Akita mice. The Akita mouse carries a mutation for the *Ins2* gene which disrupts a disulfide bond between the *α* and *β* chain of proinsulin, leading to the mis-folding of the mutated insulin, and by which the mutated insulin induces ER stress in *β* cells to cause diabetes [56].

It is likely that inflammatory cytokines produced by islet-infiltrated autoreactive immune cells are the major factors causing *β*-cell death in type 1 diabetes [57]. In the early stage of type 1 diabetes, the autoreactive immune cells such as macrophages and T lymphocytes infiltrate into the pancreatic islets along with the secretion of inflammatory cytokines such as IL-1*β*, IFN-*γ*, and TNF-*α*, which then induce ER stress to mediate *β*-cell destruction. The damaged or dying *β* cells also release danger signals such as high-mobility group box 1 and heat shock proteins (HSPs), to alert the immune system for the presence of *β*-cell injury, which in turn further promotes autoimmune progression [57–60]. Studies have shown that stimulation of *β* cells with IL-1*β* and IFN-*γ* induces the expression of death protein 5 (DP5), and through which these cytokines mediate *β*-cell apoptosis via ER stress [61]. Knockdown of DP5 provides protection for *β* cells against inflammatory cytokine-induced ER stress [61]. Insult of *β* cells with IL-1*β* and IFN-*γ* has also been found to decrease the expression of sarcoendoplasmic reticulum pump Ca^2+^ ATPase (SERCA) 2b, which controls the storage of ER Ca^2+^ [62]. It has been well demonstrated that altered ER Ca^2+^ concentration induces the accumulation of unfolded proteins in ER associated with the induction of UPR and ER stress in *β* cells [63].

Given that hyperglycemia only occurs when *β* cells fail to compensate the increased demand for insulin, *β* cells are usually “exhausted” in T1D patients [54]. Therefore, other than the ER stress induced by autoimmune response, *β* cells in T1D patients are also under ER stress caused by altered insulin synthesis. In later case, the increased insulin demand requires the remaining functional *β* cells to increase insulin synthesis to compensate the decrease of *β* mass. While this process in short term is beneficial for control of blood glucose homeostasis, it also induces ER stress, which in turn exacerbates *β*-cell dysfunction to promote disease progression and diabetes onset. Collectively, there is convincing evidence that ER stress plays an essential role in *β*-cell destruction during the course of T1D development.

## 5. The Impact of ER Stress on Modulation of Autoimmune Response

Unlike its well-defined effect on autoimmune-mediated *β*-cell destruction in type 1 diabetes, the impact of ER stress in modulating autoimmune response during the course of type 1 diabetes, however, remains poorly elucidated. There is evidence supporting that other than its critical roles played in *β*-cell destruction, ER stress also modulates the functionality of immune cells with implications in autoimmune response in type 1 diabetes.

It has been well accepted that the presence of *β*-cell-specific autoantibodies serves as a marker for the initiation and progression of autoimmunity in type 1 diabetes [64]. Studies have shown that IRE1, a key molecule in UPR, modulates the differentiation of antibody-producing B lymphocytes. Deficiency of IRE1 hampers pro-B cells differentiating into pre-B cells [65], and XBP-1, an IRE1 downstream molecule, is required for antibody production by mature B cells [66]. It was found that the engagement of B-cell receptor (BCR) induces ubiquitin-mediated degradation of BCL-6, a repressor for B-lymphocyte-induced maturation protein 1 (BLIMP1) [67], while BLIMP1 negatively regulates the expression of B-cell-lineage-specific activator protein (BSAP) [68], and BSAP is suggested to function as a repressor for XBP-1 [69]. In line with these results, B lymphocytes deficient in BLIMP1 failed to express XBP-1 in response to LPS stimulation [66].

Recent studies highlighted the importance of innate immunity in the pathogenesis of type 1 diabetes [59, 60], while elements of the UPR pathway are found to regulate innate immune response [70]. The expression of CREBH, an ER stress-associated transcription factor, can be induced by inflammatory cytokines such as IL-1*β* and IL-6, which in turn regulates the transcription of serum amyloid P-component and C-reactive protein, the two critical factors implicated in innate immune responses [71]. Furthermore, the differentiation of dendritic cells (DCs), the most critical innate immune cells, is regulated by UPR signaling element, XBP-1 [72]. High levels of mRNA splicing for XBP-1 are found in DCs, and mice deficient in XBP-1 show altered development of both conventional and plasmacytoid DCs. Loss of XBP-1 renders DCs vulnerable to ER stress-induced apoptosis [72]. Moreover, the capacity for DCs secretion of inflammatory cytokine IL-23 is regulated by CHOP, a UPR mediator. CHOP can directly bind to the *IL-23* gene and regulate its transcription. ER stress combined with Toll-like receptor (TLR) agonists was found to markedly increase the mRNA of IL-23 p19 subunit and the secretion of IL-23, while knockdown of CHOP suppressed the induction of IL-23 by ER stress and TLR signaling [73].

Richardson and coworkers reported that innate immune response induced by* P. aeruginosa* infection causes ER stress in *C. elegans*, and mutations with loss of function for XBP-1 lead to larval lethality [74]. In consistent with this result, the polymorphisms within the *XBP-1* gene were found to be associated with Crohn's disease and ulcerative colitis in humans [75], and the two autoimmune diseases share similar properties as type 1 diabetes. Loss of XBP-1 in intestinal epithelial cells induces Paneth cell dysfunction and overactive epithelium, leading to impaired mucosal defense to *Listeria monocytogenes* and increased sensitivity to colitis [75].

Other than the IRE1/XBP-1 axis, the PERK/eIF2*α*/ATF4 axis of UPR is also found to be associated with innate response. TLR signaling, the most important innate signaling pathway, is reported to induce selective suppression of the ATF-4/CHOP axis of UPR pathway [76]. TLR signaling decreases eIF2*α*-induced ATF4 translation. For example, pretreatment of LPS, an agonist for TLR4, suppressed ATF4/CHOP signaling and prevented systemic ER stress-induced apoptosis in macrophages, renal tubule cells, and hepatocytes [76]. In contrast, loss of Toll-IL-1R-containing adaptor inducing IFN-*β* (TRIF), an important adapter for TLR signaling, abrogated the protective effect of LPS on systemic ER stress-induced renal dysfunction and hepatosteatosis, suggesting that TLR signaling suppresses ATF4/CHOP via a TRIF-dependent pathway [76].

## 6. Pathways for Cytokines Induction of ER Stress

**Figure 3:**
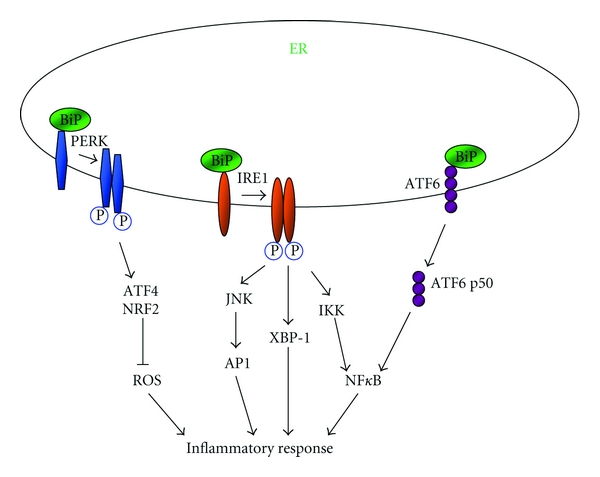
The possible implication of UPR in inflammatory response. UPR is associated with inflammation via a variety of mechanisms involving ROS, JNK, and NF*κ*B. PERK promotes ATF4 and NRF2, which then suppress ROS production by activating antioxidant pathway. Upon activation, IRE1/TRAF2 recruits IKK, leading to the phosphorylation of I*κ*B*α* and subsequent activation of NF*κ*B. IRE1/TRAF2 can also activate AP1, resulting in the activation of JNK. XBP-1 induced by IRE1 can further induce the expression of various genes implicated inflammation. Furthermore, ATF6, the other axis of UPR signaling, can promote inflammation via activating NF*κ*B.

Upon the insults of pathogens, mutated self-antigens, or tissue damage, the immune system initiates inflammatory response by releasing copious amount of cytokines. UPR and ER stress are interconnected with inflammatory cytokines through multiple mechanisms including reactive oxygen species (ROS), NF*κ*B, and JNK (Figure 3). ROS are highly reactive small molecules with unpaired electrons. They are important mediators of inflammatory response. The accumulation of ROS, referred to as oxidative stress, was confirmed to be associated with ER stress [77]. Oxidizing condition is required for the disulphide bond formation during the process of protein folding [78]. Increased protein folding load may lead to oxidative stress. The PERK axis of UPR signaling is reported to be able to activate antioxidant pathway by promoting ATF4 and nuclear factor-erythroid-derived 2-related factor 2 (NRF2) [79, 80]. Therefore, loss of PERK markedly increases ROS accumulation induced by toxic chemicals [79, 81]. The IRE1/TRAF2 axis of UPR can recruit I*κ*B kinase (IKK), leading to the activation of NF*κ*B, a key regulator in inflammation [82]. As a result, NF*κ*B activation and TNF-*α* production are reduced in cells lacking IRE1 [82]. Furthermore, the IRE1/TRAF2 axis can activate JNK, and by which it induces the expression of inflammatory genes by activating activator protein 1 (AP1) [83]. ATF6, the third axis of UPR signaling, can also activate NF*κ*B pathway, in which suppression of ATF6 reduces NF*κ*B activation caused by BiP degradation [84].

Other than the above described pathways, cytokines may also induce ER stress via inducible nitric oxide (NO) synthase (iNOS) and JNK pathway. JNK pathway is activated by IL-1*β*. Suppression of JNK by its inhibitor SP600125 protected *β* cells from IL-1*β*-induced apoptosis [85]. Cytokines have been evidenced to induce the expression of iNOS, leading to excessive NO production. Stimulation with IL-1*β* and IFN-*γ* activates ER stress pathway and induces *β*-cell apoptosis via NO synthesis [62]. NO has been suggested to be an important mediator of *β*-cell death in type 1 diabetes. Inflammatory cytokines including IL-1*β*, IFN-*γ*, and TNF-*α* can induce iNOS expression in *β* cells which then produces copious amount of NO [50]. Excessive NO induces DNA damage and thus results in *β*-cell apoptosis through p53 pathway or necrosis through poly(ADP-ribose) polymerase (PARP) pathway [54]. Moreover, NO depletes ER Ca^2+^ stores via activating Ca^2+^ channels or inhibiting Ca^2+^ pumps [86–88]. Depletion of Ca^2+^ then leads to ER stress and apoptosis in *β* cells via the induction of CHOP signaling [55, 89].

## 7. Mechanisms Underlying ER-Stress-Induced ***β***-Cell Death

ER stress is a key mediator for *β*-cell death in type 1 diabetes. The primary purpose of ER stress or UPR is to compensate the damage caused by the disturbances of normal ER function. However, continuous ER dysfunction would eventually render cells undergoing apoptosis. The mechanisms by which ER stress induces cell death are not fully elucidated, due to the fact that multiple potential participants involved but little clarity on the dominant death effectors in a particular cellular context. In general, ER stress induction of cell death can be illustrated in three phases: adaptation, alarm, and apoptosis [44].

The phase for adaptation response is initiated to restore the homeostasis of ER and to protect cells from damage induced by the disturbances of ER function. As described earlier, the signaling for UPR involves three axes of responses: IRE1, PERK, and ATF6. These axes interact between each other and form a feedback regulatory mechanism to control the activity of UPR. The accumulation of unfolded proteins in ER results in the engagement of ER-resident chaperon BiP, and as a consequence, IRE1, PERK, and ATF6 are released from BiP. Therefore, overexpression of BiP can prevent cell death induced by oxidative stress, Ca^2+^ disturbances, and hypoxia [90]. PERK is oligomerized and phosphorylated when released from BiP. Activated PERK inactivates eIF2*α* to reduce mRNA translation and protein load on ER. Therefore, PERK deficiency results in an abnormally elevated protein synthesis in response to the accumulation of unfolded proteins in ER, which renders cells highly sensitive to ER stress and ER stress-induced apoptosis [91]. Similarly, as a downstream molecule of PERK, eIF2*α* is required for cell survival upon the insult of ER stress, and a mutation at the phosphorylation site of eIF2*α* (Ser51Ala) abolishes the translational suppression in response to ER stress [52]. Similar as PERK, IRE1 becomes dimerized and activated once released from BiP. IRE1 induces XBP-1 by promoting the splicing of its mRNA [45]. XBP-1 is a transcriptional factor belonging to the bZIP-family and is responsible for the transcription of many adaptation genes implicated in UPR. Unlike PERK and IRE1, ATF6 translocates into the Golgi apparatus upon the release from BiP. The transmembrane domain of ATF6 is cleaved in the Golgi apparatus and is then relocated into the nucleus, by which it regulates gene expression [46].

During the alarm phase, many signal pathways are activated, and the expression of responsive genes has been induced to alert the system. For example, the cytoplasmic part of IRE1 binds to TNF receptor-associated factor 2 (TRAF2), a key adaptor for TNF-mediated innate immune signaling. TRAF2 would then activate NF*κ*B pathway via activating IKK and activate the signaling for c-Jun N-terminal kinases (JNK) by apoptosis signal-regulating kinase 1 (Ask1). Studies have shown that dominant negative TRAF2 suppresses the activation of JNK by IRE1 in response to ER stress [92]. Importantly, TRAF2 is also a critical component for E3 ubiquitin-protein ligase complex [93], which binds to Ubc13 and promotes the noncanonical ubiquitination of substrates. The Ubc13-dependent ubiquitination of TRAF2 is suggested to be required for the activation of JNK [94]. In addition, IRE1 can further activate JNK signaling through interacting with c-Jun N-terminal inhibitory kinase (JIK) [95].

Although the purpose for the initiation of adaptation response is to restore the homeostasis of ER, apoptosis however could occur, once the accumulation of unfolded proteins exceeds the cellular regulatory capacity. The action for apoptosis is initiated by the activation of several proteases such as caspase-12, caspase-4, caspase-2, and caspase-9. Studies in rodents provided evidence supporting that caspase-12 is involved in ER stress-induced apoptosis. Caspase-12 is activated by IRE1 upon the insult of ER stress. Mice deficient in caspase-12 are resistant to ER stress-induced apoptosis, but remain susceptible to apoptosis induced by other stimuli [96]. There is evidence that caspase-12 can also be activated by interacting with TRAF2, a signaling molecule downstream of IRE1 [95]. In response to ER stress, caspase-7 is translocated from the cytosol to the ER surface, which then activates procaspase-12 as well [97]. The human caspase-4 is the closest paralog of rodent caspase-12, which is normally located on the ER membrane. However, caspase-4 can only be activated by ER stress-inducing reagents not by the other apoptotic reagents, and knockdown of caspase-4 by siRNA reduces ER stress-induced apoptosis in neuroblastoma cells [98]. Similarly, caspase-2 and caspase-9 are found to be activated in the early phase of ER stress and inhibition of their activation either by inhibitors or siRNA reduces ER stress-induced apoptosis [99]. Studies also suggest that some members of inhibitor of apoptosis protein family prevent ER stress-induced cell death via interacting with caspase-2 and caspase-9 [99].

Other than the implication of caspases, Ask1 kinase and CCAAT/enhancer binding protein (C/EBP) homologous protein (CHOP) are also critical mediators for ER stress-induced cell death. IRE1/TRAF2 complex recruits Ask1 and activates subsequent JNK signaling. Studies have shown that the activation of JNK inhibits antiapoptotic protein BCL-2 [100] and induces proapoptotic protein Bim [101, 102]. Loss of *Ask1* suppresses ER stress-induced JNK activation and provides protection for cells against ER stress-induced death [103]. CHOP is a transcription factor belonging to basic leucine zipper transcription factor (bZIP) family. Many inducers of UPR including ATF4, ATF6, and XBP-1 up-regulate CHOP expression, and phosphorylation of CHOP at ser78 and ser81 by p38 MAPK enhances its transcriptional activity [44, 104]. Upon its activation, CHOP suppresses anti-apoptotic protein BCL-2 which in turn induces cells undergoing apoptosis [105–107].

## 8. Conclusion and Future Directions

There is convincing evidence that ER stress plays an essential role in autoimmune-mediated *β*-cell destruction. Feasible evidence also supports that ER stress modulates autoimmune response during T1D development (Table 1). ER stress in *β* cells can be either triggered by autoimmune responses against *β*-cell self-antigens and/or by the increase of compensated insulin synthesis. During the course of type 1 diabetes, autoreactive immune cells secrete copious amount of inflammatory cytokines such as IL-1*β*, TNF-*α*, and IFN-*γ* into the islet milieu, which stimulate excessive production of NO in *β* cells and mediate *β*-cell destruction by inducing ER stress. Recent studies further suggest that ER stress also modulates the functionality of immune cells with implications in autoimmune progression. The absolute insulin deficiency in T1D patients renders the residual *β* cells for compensated insulin secretion to meet the demands of insulin for maintaining blood glucose homeostasis. This increase in insulin biosynthesis could overwhelm the folding capacity of ER, leading to UPR and ER stress in *β* cells, which in turn exacerbates *β*-cell dysfunction and T1D onset.

It should be kept in mind that the mechanisms underlying autoimmune-mediated *β*-cell destruction in type 1 diabetes are complex, and ER stress is unlikely the exclusive mechanism implicated in disease process. Despite recent significant advancement in this field, there are still many questions yet to be addressed. Can ER stress be served as a biomarker for *β*-cell destruction and autoimmune progression in the clinic setting? Are there additional factors for induction of ER stress in *β* cells during T1D development? Does modulation of ER stress in immune cells attenuate autoimmune progression? Does blockade of ER stress protect *β* cells from autoimmune-mediated destruction? Future studies aimed at dissecting these questions would provide a broadened insight for T1D pathogenesis and would have great potential for developing novel therapeutic strategies against this devastating disorder. 

## Figures and Tables

**Table 1:** Publications relevant to ER stress in the regulation of immune response and *β*-cell destruction.

Author	Defective/mutant gene	Species	Major finding	Reference
Harding et al.	PERK^−/−^	Mouse	PERK-deficient mice are extremely susceptible to diabetes. They display a progressive *β*-cell loss and hyperglycemia with aging.	[49]
Delépine et al.	PERK^−/−^	Human	Deficiency of PERK in human results in Wolcott-Rallison syndrome, which is characterized by early infancy insulin-dependent diabetes and multisystemic dysfunction.	[50]
Scheuner et al.	eIF2*α* mutant (Ser51Ala)	Mouse	Ser51Ala mutation of eIF2*α* shows a deficiency in pancreatic *β* cells manifested by the smaller core of insulin-secreting *β* cells and attenuated insulin secretion, and the mice die from hypoglycemia at their early infancy.	[52]
Ron et al.	Ins2 mutation	Mouse	Ins2 mutation in Akita mice disrupts disulfide bond between the *α* and *β* chain of proinsulin, which leads to the mis-folding of the mutated insulin and further induces ER stress in *β* cells and diabetes.	[56]
Zhang et al.	IRE1^−/−^	Mouse	Pro-B cells failed to differentiate into pre-B cells when deficient for IRE1.	[65]
Iwakoshi et al.	XBP-1^−/−^	Mouse	Deficiency of XBP-1 results in the impacted development of both conventional and plasmacytoid DCs. Loss of XBP-1 renders DCs vulnerable to ER stress-induced apoptosis.	[72]
Goodall et al.	CHOP knockdown		Knockdown of CHOP suppressed the production of IL-23 induced by ER stress and TLR signaling.	[73]
Richardson et al.	XBP-1 mutation	*C. elegans*	Innate immune response induced by *P. aeruginosa* infection causes ER stress in *C. elegans*, and mutations with loss of function for XBP-1 lead to larval lethality.	[74]
Kaser et al.	XBP-1 polymorphisms	Human	Loss of XBP-1 in intestinal epithelial cells induces Paneth cell dysfunction and overactive epithelium, leading to impaired mucosal defense to Listeria monocytogenes and increased sensitivity to colitis, an inflammatory disease sharing similar properties with T1D. The polymorphisms within the XBP-1 gene are associated with Crohn's disease and ulcerative colitis in humans.	[75]
Nakagawa et al.	Caspase-12^−/−^	Mouse	Caspase-12 is involved in ER stress-induced apoptosis. Mice deficient in caspase-12 are resistant to ER stress-induced apoptosis, but remain susceptible to apoptosis induced by other stimuli.	[96]
Hitomi et al.	Caspase-4 knockdown	Human	Human caspase-4, the closest paralog of rodent caspase-12, is involved in ER stress-induced apoptosis. Knockdown of caspase-4 by siRNA reduces ER stress-induced apoptosis.	[98]
Nishitoh et al.	Ask1^−/−^	Mouse	Loss of Ask1 suppresses ER stress-induced JNK activation and protects cells from ER stress-induced death.	[103]

## Abbreviations

AP1: Activator protein 1
Ask1: Apoptosis signal-regulating kinase 1
ATF6: Activating transcription factor 6
BCR: B-cell receptor
BLIMP1: B-lymphocyte-induced maturation protein 1
BSAP: B-cell-lineage-specific activator protein
bZIP: Basic leucine zipper transcription factor
C/EBP: CCAAT/enhancer binding protein
CHOP: C/EBP homologous protein
CPA: Cyclopiazonic acid
CREBH: Cyclic-AMP-responsive-element-binding protein H
DC: Dendritic cell
DP5: Death protein 5
eIF2*α*: Eukaryotic initiation factor 2*α*
ER: Endoplasmic reticulum
ER stress: Endoplasmic reticulum stress
ERAD: ER-associated degradation
ERSE: ER stress enhancer
HMGB1: High-mobility group box 1
HSPs: Heat shock proteins
IKK: I*κ*B kinase
iNOS: Inducible nitric oxide synthase
IRE1: Inositol-requiring enzyme 1
IRS-1: Insulin receptor substrate-1
JIK: c-Jun N-terminal inhibitory kinase
JNK: c-Jun N-terminal kinases
NO: Nitric oxygen
NRF2: Nuclear factor-erythroid-derived 2-related factor 2
PARP: Poly(ADP-ribose) polymerase
PERK: Pancreatic endoplasmic reticulum kinase
RER: Rough endoplasmic reticulum
ROS: Reactive oxygen species
SER: Smooth endoplasmic reticulum
SERCA: Sarcoendoplasmic reticulum pump Ca^2+^ ATPase
SR: Sarcoplasmic reticulum
T1D: Type 1 diabetes
TLR: Toll-like receptor
TRAF2: TNF receptor-associated factor 2
TRIF: Toll-IL-1R-containing adaptor inducing IFN-*β*
UPR: Unfolded protein response
UPRE: Unfolded protein response element
XBP-1: X box protein-1.
